# Impact of Nisin on Proliferation of Background Microbiota, Pressure-Stressed and Wild-Type *Listeria monocytogenes*, and *Listeria innocua* During a Real-Time Shelf-Life Study

**DOI:** 10.3390/microorganisms13030668

**Published:** 2025-03-15

**Authors:** Ranju Kafle, Aliyar Cyrus Fouladkhah

**Affiliations:** 1 Public Health Microbiology Laboratory, Tennessee State University, Nashville, TN 37209, USA; 2 Public Health Microbiology Foundation^SM^, Nashville, TN 37027, USA

**Keywords:** *Listeria monocytogenes* surrogate, mesophilic background microbiota, pressure-stressed *Listeria* spp.

## Abstract

With the rapid implementation of high-pressure processing in many sectors of the food industry, considerations associated with pressure-stressed microorganisms are emerging. Nisin was utilized in this study for controlling the proliferation of *Listeria monocytogenes* and *L. innocua* inoculated on cold-smoked trout during a 4-week refrigerated shelf-life trial. Wild-type and pressure-stressed phenotypes of *Listeria* were compared in this study. The pressure-stressed phenotypes were prepared by treating the surrogate strain and pathogen mixture at 103.4 MPa (15K PSI) for 20 min. *L. monocytogenes* multiplied extensively during the 4-week refrigerated trial and counts were increased (*p* < 0.05) from 3.68 ± 0.1 log CFU/g on the first week to 6.03 ± 0.1 log CFU/g. Both phenotypes and the surrogate microorganisms illustrated similar (*p* ≥ 0.05) multiplication trends. Unlike samples subjected to water treatment, nisin was effective (*p* < 0.05) in keeping the microbial counts lower compared with the controls, particularly earlier during the shelf-life trial. Our study illustrates that the selected surrogate microorganism has comparable sensitivity to nisin relative to *L. monocytogenes* and thus could be used interchangeably in future public health microbiology challenge studies with similar scope. Additionally, we observed that pressure-stressed *L. monocytogenes* has proliferation and sensitivity to nisin comparable to wild-type pathogen.

## 1. Introduction

*Listeria monocytogenes* was recognized as a prominent pathogen upon its isolation during an epidemic of listeriosis in newborns in Germany in 1949 [1]. Although there are twenty-eight species in the *Listeria* genus, only two (*monocytogenes*, and *ivanovii*) can cause disease in humans and animals [2]. According to the U.S. Centers for Disease Control and Prevention, in a typical year, *L. monocytogenes* is the third leading cause of foodborne death, accounting for close to 1600 illness episodes and more than 250 annual deaths, with a mortality rate of 15.9% in the United States [3]. Of particular importance is the fact that more than 98% of Listeriosis cases in the United States are linked to contaminated food [4]. After consuming contaminated food, *L. monocytogenes* can travel from the human gut to various target organs through blood. The pathogen can target the liver to multiply and after proliferation in the liver, persistent cases of bacteremia and secondary invasion of other target organs like the brain and gravid uterus can occur [5,6].

One of the main sources of *Listeria* spp., and specifically *L. monocytogenes*, is ready-to-eat (RTE) food products. In a multiyear epidemiological study of RTE producers under inspection in the United States, it was observed that 71% of the facilities had at least one positive result of *Listeria* spp. for samples obtained from their food and/or food-contact surfaces [7]. Although it is noteworthy that there may or may not be a direct correlation between the presence of *Listeria* spp. and *L. monocytogenes*, the above-mentioned epidemiological study indicated that, overall, 4.9% of the samples were positive for *Listeria* spp., illustrating the ubiquitous nature of this important microorganism [7]. A Canadian outbreak in 2023 linked to recalled plant-based refrigerated beverages resulted in 20 confirmed cases, 15 hospitalizations, and three deaths, and a recent outbreak linked to deli meat initiated from the U.S. State of Virginia was linked with at least 10 deaths and 60 hospitalizations, further emphasizing the public health importance of this pathogen [8,9]. Various investigations have reported *L. monocytogenes* in RTE food products worldwide, with considerable variations for products and regions of the world. In Belgium, as an example, the prevalence of *Listeria* in RTE food products was 6.7% in deli salads with mayonnaise, 1.1% in precooked meat products, and 22% for smoked fish [10,11,12,13]. Among various commodities, a range of seafood products, such as marinated fish, cooked and frozen seafood, and smoked fish, are of particular importance and have been associated with the *Listeria* outbreaks. This highlights the challenge of controlling *Listeria* in seafood due to its ability to withstand diverse environmental stressors and multiply under refrigerated temperatures [14].

Cold-smoked rainbow trout (*Oncorhynchus mykiss*) is an important vehicle for this pathogen in the food chain [15,16,17]. *Listeria* can multiply to hazardous levels during storage in refrigerated conditions; therefore, health complications are possible even if the contamination level is less than 100 CFU/g of the product [18]. *L. monocytogenes* shows a higher multiplication rate in fish, compared to beef and chicken at 4 °C and under aerobic conditions [19].

The use of *L. monocytogenes* in commercial food processing facilities is prohibited due to its pathogenic nature since there are strict requirements for Biosafety Level II (BSL-2) facilities that conduct experiments involving *L. monocytogenes* because of safety concerns [20]. Thus, the ideal substitute is a surrogate bacterium that shares the common physiological traits of the pathogenic bacterium [21]. *L. innocua* is a nonpathogenic surrogate for *L. monocytogenes*, frequently found in environments similar to *L. monocytogenes* [22].

Although traditional preservation methods such as thermal preservation methods alter the taste, flavor, and nutritional quality of the products, they have been utilized by the food industry for decades. As a result, non-thermal methods of food preservation with minimal impact on the quality and organoleptic properties of food are gaining popularity. High-pressure processing (HPP) is a potential substitution for a thermal technique that transfers pressure instantly and uniformly throughout the sample without compromising the quality of the food sample [23,24]. HPP alters the morphology and physiology of bacteria, including membrane permeability and protein denaturation. As a result, this disruption can cause cellular damage or death, impacting vital processes and leaking cellular content. It also affects membrane proteins, protein structure, and ATP synthesis of bacteria [25]. However, limited information is available in the literature regarding the fate and multiplication of pressure-stressed microorganisms, i.e., those surviving treatment with elevated hydrostatic pressure.

A highly effective bacteriocin, nisin, produced by *Lactococcus lactis*, can be used to ensure the safety of ready-to-eat commodities. More than 40 countries use nisin as a preservative, as it is approved by the US Food and Drug Administration (FDA) and the European Food Safety Authority. Nisin attributes broad-spectrum antimicrobial properties, and Gram-positive bacteria are especially susceptible to its effect [26]. Nisin has demonstrated efficiency against a diverse range of Gram-positive bacteria and spoilage microorganisms, including *Staphylococcus aureus*, *L. monocytogenes*, and *Clostridium* spp. [27] and in a variety of foods such as dairy products and desserts, fish and seafood, fruit juices, beverages, and veterinary medicine for the treatment of bovine mastitis [28].

The current study is a 4-week shelf-life study to investigate the fate and proliferation of *L. monocytogenes* on cold-smoked trout during aerobic refrigerated storage. The impact of nisin during the trial was additionally investigated against wild-type and pressure-stressed phenotypes of the pathogen. The fate and proliferation of *L. innocua* as a surrogate for *L. monocytogenes* were additionally investigated in this study.

## 2. Materials and Methods

### 2.1. Preparation of Listeria Strains and Inocula

The frozen stock of five *L. monocytogenes* strains used in our experiment were those with ATCC^®^ numbers 51772^TM^ (serotype 1/2a), 51779^TM^ (serotype 1/2c), BAA-2658^TM^ (serotype 1/2b), 13932^TM^ (serotype 4b), BAA-751^TM^ (serotype 1/2b), and a non-pathogenic strain of *L. innocua* (ATCC^®^ 33090^TM^, serotype 6a), preserved at −80 °C in glycerol. Strains that represent various lineages and ribotypes were chosen based on public health and the food industry’s significance and based on previously published work of the public health microbiology laboratory of Tennessee State University. Each strain was separately transferred into 10 mL Tryptic Soy Broth (Difco, Becton Dickinson, Franklin Lakes, NJ, USA) containing 0.6% yeast extract (TSB + YE) and incubated at 37 °C for 22–24 h. After incubation, the bacterial suspension was homogenized using a high-speed vortex (Scientific Industries, Bohemia, NY, USA, Model SI-0236), and 0.1 mL was transferred into another 10 mL TSB + YE and was then incubated again at 37 °C for 22–24 h. After incubation and homogenization, the cells were plated onto the surface of Tryptic Soy Agar (Difco, Becton Dickinson, Franklin Lakes, NJ, USA) containing 0.6% yeast extract (TSA + YE) using a sterilized inoculation loop and incubated at 37 °C for 22–24 h to obtain individual colonies. These plates were then stored in a 4 °C refrigerator for up to one month prior to the trial.

Two days before the experiment, for activation of the microorganisms, a loop-full of bacteria sourced from a single colony of the above-mentioned plates, for each strain separately, was transferred into 10 mL of TSB + YE and incubated at 37 °C for 22–24 h. After this activation and for sub-culturing, 0.1 mL (each strain separately) was pipetted aseptically into another fresh sterilized 10 mL of TSB + YE and incubated again at 37 °C for 22–24 h. After incubation, the overnight bacterial suspension of each strain was used to harvest the cells through centrifugation for 15 min at 6000 revolutions per min twice (Eppendorf North America, Hauppauge, NY, USA; Model 5424, Rotor FA-45-24-11). After the first round of centrifugation, the supernatant, containing cell components, secondary metabolite, and growth media, was discarded, and the microbial pellet was re-suspended in PBS (VWR International, Radnor, PA, USA). The process was repeated to further purify the bacterial inocula, and after the second re-suspension of the strains in PBS (VWR International, Radnor, PA, USA), the five strains of the pathogen were composited into an inoculum to ensure equal representation of all strains.

### 2.2. Sample Preparation and Inoculation

Farm-raised cold-smoked rainbow trout (*Oncorhynchus mykiss*) was obtained from a local supermarket in Nashville, Tennessee, with a sodium content of aproximately 1020 mg/100 g. The product was marketed as a “natural” commodity without any listed antimicrobials such as nitrite or nitrate. The skinless samples were then cut aseptically into 5.0 ± 0.1 g portions, and each sample was placed inside a sterile petri dish (VWR International, Radnor, PA, USA). Samples were then randomly assigned to each of the four inoculation groups of (i) a five-strain mixture of wild-type *L. monocytogenes*, (ii) a five-strain mixture of pressure-stressed *L. monocytogenes*, (iii) one strain of wild-type *L. innocua*, and (iv) one strain of pressure-stressed *L. innocua*. Each of these four was additionally divided into three treatment sections of (a) no treatment (untreated control), (b) with 0.1 mL of deionized and sterilized water added (treated control), and (c) with 5000 IU of nisin. This concentration of nisin was selected based on preliminary trials to ensure efficacy and using the antimicrobial at a common level of use in the food industry [29]. Pathogen inocula were then 10-fold serially diluted in PBS for a target inoculation level of 3 to 4 log CFU/g. Samples were stored aerobically to mimic consumers’ and food service handling of the product after opening the package and storing the commodity at refrigeration prior to consumption.

The powdered nisin (Sigma-Aldrich, St. Louis, MO, USA) was used at a concentration of 5000 IU/5.0 g of samples, with 1000 IU being equivalent to 0.025 mg of nisin [29]. The International Unit (IU) of nisin is defined as the quantity of nisin required to inhibit a single *Streptococcus agalactiae* cell in 1 mL of broth [29]. Before the addition of bacteriocin, nisin was transferred into Phosphate-Buffered Saline (PBS, VWR 135 International, Radnor, PA, USA), and the insoluble solids were removed by centrifuging (Eppendorf North America, Hauppauge, NY, USA; Model 5424, Rotor FA-45-24-11) at the intensity of 3000 revolutions per min for one min. After centrifuging, the supernatant was filter-sterilized and inoculated onto the surface of fish samples. The inoculated bacteriocin was then spread evenly into the surface of the product using a sterilized glass spreader prior to the inoculation of the above-mentioned four inocula.

### 2.3. Preparation of Pressure-Stressed Microbial Cells

A mixture of 1.5 mL of *L. monocytogenes* and a single strain of *L. innocua* was transferred to the PULSE tubes without a disk (Pressure Bioscience Inc., South Easton, MA, USA) inside PBS (VWR International, Radnor, PA, USA) at target bacterial load of 7 to 8 log CFU/mL. The PULSE tubes were then treated at 4 °C and at an elevated hydrostatic pressure of 103.4 MPa (15K PSI) for 20 min to yield the target bacterial count of 6 log CFU/mL after treatment [30]. The HUB Explorer PBI software Version 2.3.11 (Pressure Bioscience Inc., South Easton, MA, USA) was used to monitor and automatically record the temperature every 3 s. The transmission fluid for the pressure treatment was distilled water (<30 ppm total dissolved solids). Residual air from the chamber was purged using a pump on chamber closure to ensure treatments were hydrostatic in nature. The temperature was monitored using a K-type thermocouple (Omega Engineering Inc., Norwalk, CT, USA) mounted inside the chamber wall, secured with thermal paste (Model 5 AS5-3.5G, Arctic Silver, Visalia, CA, USA), and connected to HUB Explorer PBI software. The temperature was regulated by a refrigerated circulating water bath (Model 160s, VWR International, Radnor, PA, USA) mechanically connected to a stainless-steel jacket surrounding the pressure-processing chamber.

### 2.4. Microbiological and Physiochemical Analysis

On each day of the trial, samples were aseptically transferred into the sterile filtered stomaching bag (Whirl-Pak, Modesto, CA, USA) and 20 mL of sterile Dey/Engley neutralizing broth (D/E Broth; Difco, Becton Dickinson, Franklin Lakes, NJ, USA) was added to each bag followed by mastication for 2 min at 200 revolutions per min using a Triple Mix Paddle Blender (Boekel Scientific, Feasterville-Trevose, PA, USA) to achieve an even distribution of microorganisms within the suspension. Samples were then 10-fold serially diluted in 0.1% Maximum Recovery Diluent (MRD; Difco, Becton Dickinson, Franklin Lakes, NJ, USA) and were spread-plated onto the surface of selective and non-selective media. The selective medium, i.e., Polymyxin Acriflavin Lithium-chloride Ceftazidime Esculin Mannitol (PALCAM) Agar was supplemented with Ceftazidime (Becton, Dickinson and Company, Sparks, MD, USA) and non-selective medium of Tryptic Soy Agar (Difco, Becton Dickinson, Franklin Lakes, NJ, USA) was supplemented with 0.6% yeast extract.

After 48 h of incubation at 37 °C, the bacterial colonies were counted manually using the Quebec Colony Counter based on the U.S. Food and Drug Administration Bacteriological Analytical Method [31]. Counts of non-selective medium after incubation at 37 °C were reported as mesophilic background microbiota (mesophilic aerobic plate count). A water activity meter (Lab Swift-aw water activity meter, Novasina, Lachen, Switzerland) was also used to measure the water activity of the samples every week. The pH of the samples was similarly monitored weekly using a calibrated pH meter (S400-kit SevenExcellence, Mettler Toledo, Columbus, OH, USA).

### 2.5. Design and Data Analysis

This study involved two microbiologically independent replicates, serving as blocking factors within a randomized complete block design. Treatments were implemented across two distinct blocks, each comprising two replications. Each of these replications was additionally repeated twice as two microbiological repetitions and microbial analyses were conducted on selective and non-selective media. Thus, each presented value is the mean of eight independent observations (2 blocks, 2 replications, 2 microbiological repetitions). The microbial counts were log-transformed in Microsoft Excel (Microsoft Corp, Redmond, WA, USA), and the data were then imported to SAS version 9.4 software (SAS Institute Inc, Cary, NC, USA) for further inferential and descriptive statistics. Initially, the log-normality of the data was confirmed by running diagnostics using ods graphics in the General Linear Model (GLM) procedure of SAS version 9.4. After the confirmation of log normality and homogeneity of variances (Levene’s test with *p* ≥ 0.05), the GLM procedure was used for mean separations using Tukey-adjusted (expressed by alphabet letters in figures) and Dunnett’s adjusted (expressed by “*” in figures) analyses of variance. In the former analysis, all pair-wise comparisons were considered with the largest value receiving the letter “A” (for selective counts) and “a” (for non-selective counts), while the latter only compared each treatment with the control, thus, bars marked by “*” are statistically (*p* < 0.05) different than the control. For both Tukey- and Dunnett-based ANOVA, two separate analyses were conducted for selective and non-selective media.

## 3. Results and Discussion

The temperature of the samples remained unchanged (*p* ≥ 0.05) during the course of the 4-week shelf-life trial. Temperature values (°C, mean ± standard deviation) for the weeks 0 to 4 were 5.23 ± 0.1, 5.08 ± 0.1, 5.28 ± 0.2, 4.75 ± 0.4, and 5.33 ± 0.2, respectively. Similarly, the pH (mean ± standard deviation) of control and samples treated with distilled water (DW), and nisin were similar (*p* ≥ 0.05) and for samples of weeks 0 to 4 were 6.55 ± 0.0, 6.63 ± 0.1, 6.63 ± 0.2, 6.68 ± 0.1, and 6.72 ± 0.5, respectively. The water activity of samples without treatment (control), treated with distilled water, and nisin were also similar and remained unchanged (*p* ≥ 0.05) until week 3. Samples of week 4 exhibited lower (*p* < 0.05) water activity relative to the other weeks of the trials. The water activity (mean ± standard deviation) of samples of the weeks 0 to 4 were 0.99 ± 0.0, 0.99 ± 0.0, 0.97 ± 0.0, 0.99 ± 0.0, and 0.96 ± 0.0, respectively.

### 3.1. Fate of Wild-Type and Pressure-Stressed Listeria monocytogenes and Background Microbiota of Smoked Trout During 4-Week Aerobic Refrigerated Storage as Affected by Nisin

**Figure 1:**
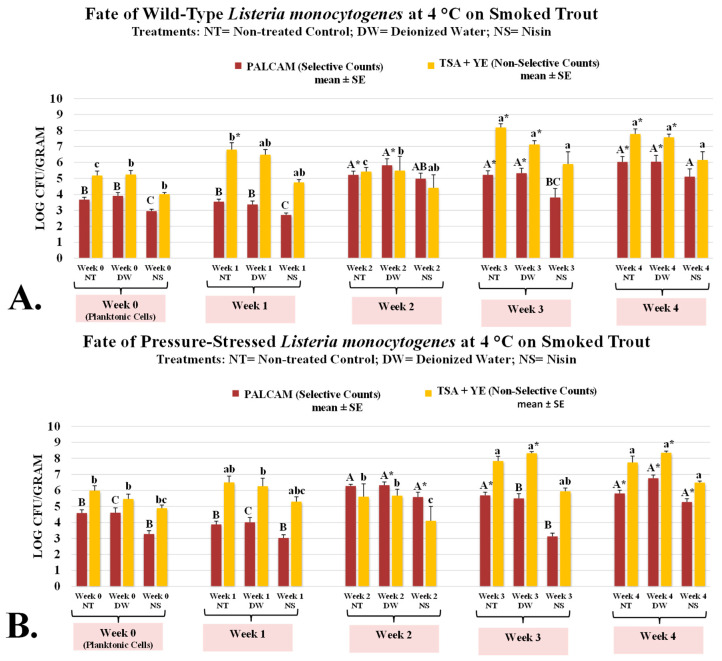
Bacteriostatic/bactericidal activity of nisin against wild-type and pressure-stressed *Listeria monocytogenes* (PALCAM counts) and mesophilic background microbiota (TSA + YE counts) during shelf-life of smoked trout. (**A**) Counts of wild-type *L. monocytogenes*. (**B**) Counts of pressure-stressed *L. monocytogenes*. PALCAM counts marked by different uppercase letters are statistically different (*p* < 0.05) from each other (Tukey-adjusted ANOVA). TSA + YE counts marked by different lowercase letters are statistically different (*p* < 0.05) from each other (Tukey-adjusted ANOVA). For each media separately, columns marked by “*” are statistically different (*p* < 0.05) than the control (Dunnett’s-adjusted ANOVA).

*L. monocytogenes* belongs to a small group of foodborne pathogens of public health concern with the capability of survival and multiplication at refrigerated temperatures [32,33]. Considering the low infective dose of the pathogen, the fate of the microorganism thus is of particular concern for ready-to-eat commodities [33,34]. Our study illustrates both selective and non-selective counts (Figure 1), representing the counts of inoculated pathogen and background microbiota (mesophilic aerobic plate count), respectively. On the day of inoculation (day 0), counts of *L. monocytogenes* were 3.68 ± 0.1 log CFU/g and these counts remained unchanged (*p* ≥ 0.05) for samples with added distilled water (DW, treated control) and were reduced (*p* < 0.05) for samples containing 5000 IU of nisin (Figure 1A). The corresponding counts with samples with DW (treated control) and nisin on this day were 3.90 ± 0.2 and 2.95 ± 0.1 log CFU/g, respectively (Figure 1A). Counts of background microbiota (mesophilic aerobic plate count) were similarly affected on week 0 (inoculation day). These counts for untreated control, those treated with DW (treated control), and nisin were 5.18 ± 0.3, 5.24 ± 0.3, and 4.02 ± 0.1 log CFU/g, respectively, with counts of samples containing nisin exhibiting more than one log (i.e., >90%) lower (*p* < 0.05) microbial counts, relative to the other two (Figure 1A). After one week of refrigerated aerobic storage counts of untreated and treated controls and those containing nisin had remained similar (*p* ≥ 0.05) and further showing similarity with trends observed on inoculation day (Figure 1A). However, the counts of background microbiota (mesophilic aerobic plate count) increased to a great extent during the one-week aerobic refrigerated storage. The mesophilic background microbiota counts of untreated and treated controls and nisin-containing samples were 6.80 ± 0.1, 6.48 ± 0.2, and 4.75 ± 0.1 log CFU/g, respectively (Figure 1A). Although early in the shelf-life, the addition of nisin exhibited promising potential for mitigating (*p* < 0.05) the counts of both pathogen and mesophilic background microbiota, this impact faded away as the shelf-life study progressed (Figure 1A). This illustrates the importance of preventive measures rather than solely relying on antimicrobials for ensuring the safety of a commodity. As an example, on the last day of the trial, the *L. monocytogenes* counts of untreated and treated controls and nisin-containing samples were 6.03 ± 0.3, 6.05 ± 0.4, and 5.11 ± 0.5 log CFU/g, respectively (Figure 1A). The corresponding counts for mesophilic background microbiota on the same week of the trial were higher and were 7.79 ± 0.3, 7.58 ± 0.2, and 6.15 ± 0.5 log CFU/g, respectively (Figure 1A).

It is also of great importance to note that *L. monocytogenes* could extensively multiply during the 4-week aerobic refrigerated shelf-life trial. The *L. monocytogenes* counts without and with nisin on week 0 were 3.68 ± 0.1, and 2.95 ± 0.1 log CFU/g, respectively (Figure 1A). By weeks 2 and 4, *L. monocytogenes* counts of samples without nisin increased (*p* < 0.05) by 1.54 and 2.35 logs, respectively. The corresponding log increases for samples with nisin were 2.03 and 2.16 logs (Figure 1A). *L. monocytogenes* is a prevalent pathogen in the smoked fish industry, a cross-sectional study, as an example, illustrated that as high as 3.8% of raw fish and 1.3% of samples obtained from four smoked fish processing plants were contaminated with this pathogen [35]. A more recent epidemiological study in Europe, although limited in sample size and scope, showed a prevalence of *L. monocytogenes* as high as 18.9% in raw and smoked samples [36]. In light of these concerns, our study illustrates that relying on antimicrobial treatment alone might not be sufficient, and a more holistic approach in the context of multiple hurdle technology should be considered to ensure the safety of these high-risk products [30,37]. The hurdle technology concept proposes the application of interventions in sequence and in combination, such as the utilization of thermal and/or non-thermal processing synergized with antimicrobial treatments, to ensure the safety of the product [37,38,39]. The application of novel technologies, such as the use of elevated hydrostatic pressure, could be a very efficacious intervention for inactivation of the pathogens [33,40]. However, any application should be carefully considered to ensure the safety and effectiveness of the treatment and its impact on the sensory and quality characteristics of the product. A recent potential challenge to the use of high-pressure processing is the concerns associated with pressure-stressed and pressure-adopted strains [41]. Currently, the literature available about the fate and proliferation of pressure-stressed microorganisms, i.e., those surviving the high-pressure treatment with or without sublethal injuries, is limited. As such, in addition to the wild-type pathogen, this study investigated the fate and proliferation of pressure-stressed *L. monocytogenes* during the refrigerated shelf-life trials (Figure 1B). Under the conditions of our experiment, pressure-stressed *L. monocytogenes* was able to survive and multiply under refrigeration (Figure 1B).

This phenotype of the *L. monocytogenes* not only remained detectable during the trial, but as well the pressure-stressed pathogen was able to multiply significantly (*p* < 0.05) during the trial by > one log CFU/g (Figure 1B). These counts on week 0 to 4 were 4.59 ± 0.2, 3.88 ± 0.2, 6.28 ± 0.1, 5.69 ± 0.2, and 5.81 ± 0.2 log CFU/g, respectively (Figure 1B). Not only the pressure-stressed phenotype of the pathogen multiplied extensively under refrigeration, the rate of multiplication for pressure-stressed pathogen (Figure 1B) was comparable with the wild-type microorganism (Figure 1A). As an example, counts of untreated controls for wild-type *L. monocytogenes* on weeks 3 and 4 were 5.22 ± 0.3 and 6.03 ± 0.3 log CFU/g, respectively (Figure 1A). The corresponding counts for pressure-stressed *L. monocytogenes* on weeks 3 and 4 were 5.69 ± 0.2 and 5.81 ± 0.2 log CFU/g, respectively (Figure 1B). The pressure-stressed cells also showed similar sensitivity to nisin, relative to wild-type cells. As an example, on week 0 pressure-stressed *L. monocytogenes* counts of untreated and treated control samples were 4.59 ± 0.2, and 4.61 ± 0.3 log CFU/g, respectively, (Figure 1B). For the same pathogen phenotype and the same week, counts of nisin-containing samples were appreciably less (*p* < 0.05) and were 3.25 ± 0.2 log CFU/g (Figure 1B). This indicates the application of nisin on week 0 was able to reduce (*p* < 0.05) more than 90% of the pressure-stressed pathogen, compared to the controls (Figure 1B). Similar to the trends observed for the wild-type phenotype of the pathogen, bactericidal and bacteriostatic impacts of nisin faded as the days of storage increased. As an example, on the last day of the shelf-life trial counts of untreated and treated controls and nisin-containing pressure-stressed pathogen were statistically nonsignificant (*p* ≥ 0.05) and were 5.81 ± 0.2, 6.76 ± 0.2, and 5.28 ± 0.2 log CFU/g, respectively (Figure 1B). These findings highlight the importance of preventive measures and application of multiple hurdle technology, as discussed earlier, rather than solely relying on one antimicrobial.

### 3.2. Fate of Wild-Type and Pressure-Stressed Listeria innocua on Smoked Trout During 4-Week Aerobic Refrigerated Storage as Affected by Nisin

**Figure 2:**
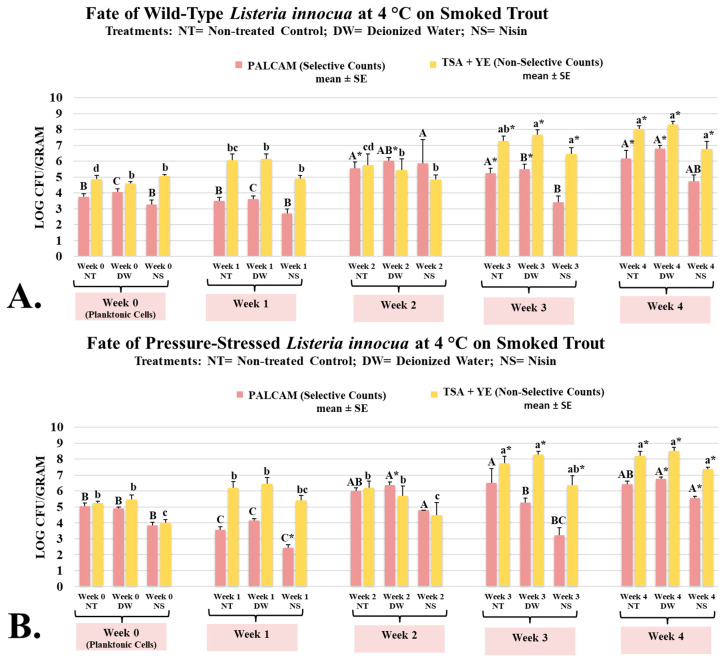
Bacteriostatic/bactericidal activity of nisin against wild-type and pressure-stressed *Listeria innocua* (PALCAM counts) and mesophilic background microbiota (TSA + YE counts) during shelf-life of smoked trout. (**A**) Counts of wild-type *L. innocua*. (**B**) Counts of pressure-stressed *L. innocua*. PALCAM counts marked by different uppercase letters are statistically different (*p* < 0.05) from each other (Tukey-adjusted ANOVA). TSA + YE counts marked by different lowercase letters are statistically different (*p* < 0.05) from each other (Tukey-adjusted ANOVA). For each media separately, columns marked by “*” are statistically different (*p* < 0.05) than the control (Dunnett’s-adjusted ANOVA).

*L. innocua* is typically considered a microorganism that does not cause human infections, and due to genetic similarities and ecological co-habitation, this microorganism could be considered a surrogate and safe alternative for *L. monocytogenes* [42]. However, the use of *L. innocua* as a surrogate for *L. monocytogenes* requires conducting a validation study to ensure the two have comparable fate, proliferation, and sensitivity to treatments [43]. Thus, the current study investigated the impact of nisin on the fate and proliferation of wild-type and pressure-stressed *L. innocua* and *L. monocytogenes*. On week 0 (planktonic cells after inoculation), *L. innocua* wild-type counts of untreated and treated controls and nisin-containing samples were 3.75 ± 0.2, 4.05 ± 0.2, and 3.26 ± 0.3 log CFU/g, respectively (Figure 2A). The corresponding counts for *L. innocua* pressure-stressed phenotype were 5.05 ± 0.2, 4.91 ± 0.1, and 3.84 ± 0.2 log CFU/g, respectively (Figure 2B). Both phenotypes of *L. innocua* were able to multiply extensively during the aerobic refrigerated shelf-life trial. Counts of wild-type *L. innocua* on weeks 0 to 4 were 3.75 ± 0.2, 3.51 ± 0.2, 5.54 ± 0.4, 5.24 ± 0.3, and 6.18 ± 0.5 log CFU/g, respectively (Figure 2A). The corresponding counts of pressure-stressed *L. innocua*, were 5.05 ± 0.2, 3.57 ± 0.2, 6.01 ± 0.2, 6.52 ± 0.9, and 6.42 ± 0.2 log CFU/g, respectively (Figure 2B). Similar to information discussed in the previous section for *L. monocytogenes*, our results, thus, illustrate that pressure-stressed phenotype of *L. innocua* not only can tolerate cold storage after exposure to elevated hydrostatic pressure, but as well could multiply under refrigeration even in presence of background microbiota (mesophilic aerobic plate count) and under low temperatures.

Under the conditions of our experiment, we observed great similarity between fate, proliferation, and sensitivity to nisin comparing *L. innocua* and *L. monocytogenes*. This is in harmony with a previously conducted studies that supported the use of *L. innocua* as a surrogate for *L. monocytogenes* in some hurdle microbiological validation studies [44]. However, some studies, in contrast, indicate that *L. innocua* should not be automatically considered a surrogate for *L. monocytogenes* and such inference should be made only after the conduct of validation studies [43]. Thus, our results propose the use of *L. innocua* as a surrogate for *L. monocytogenes* only in conditions similar to the scope of our study.

As discussed earlier in the methods section, it is important to emphasize that the current study was designed to mimic contamination of this product after processing and when the packages are opened by consumers or food service operations where there is a chance of introduction of the pathogen to the product from the environment while samples are further stored aerobically at refrigeration temperatures. Similar to the results presented in the current study, a review of the literature illustrates that nisin has great potential for inhibiting the multiplication of *Listeria* spp. As an example, in a 60-day shelf-life study conducted on smoked salmon, nisin was efficacious in reducing *L. monocytogenes* counts at the beginning and late stages of the trial [45]. In another shelf-life trial, after 34 days, up to 1.7 log reduction of *L. monocytogenes* was observed in cold-smoked salmon due to the application of nisin [46], illustrating the bactericidal properties of this bacteriocin against this ubiquitous pathogen of public health concern. Others have also illustrated that while nisin is an effective bioactive compound against *L. monocytogenes*, the impact of this antimicrobial could vary based on the strain and lineage of the pathogen [47]. In one study conducted on cold-smoked salmon, it was concluded that serotype 1/2b of *L. monocytogenes* could be more susceptible to nisin relative to 1/2a and 4b serotypes [48]. A successful validation study, thus, should include a diverse selection of strains as a cocktail for inoculation, similar to the approach used in this study, rather than relying on a single-strain inoculum. This ensures external validity and generalizability of the results.

The application of nisin as an additional hurdle in the context of hurdle technology could benefit consumers and the food industry [49]. Nisin not only could provide protection for the product during the shelf-life but could also augment the decontamination efficacy of thermal and non-thermal processing due to their similarity in mechanisms of action. Similar to high-pressure processing, as an example, that primarily disrupts the function of bacterial cell membranes [50], nisin also targets the function of bacterial membranes [27], hence, could synergistically augment the efficacy of existing hurdle(s) in manufacturing of ready-to-eat commodities.

## 4. Conclusions

Under the conditions of our experiment, we observed *L. monocytogenes* could multiply extensively during the aerobic refrigerated shelf-life study. The addition of nisin was efficacious in reducing the pathogen by more than 90% early during the storage period. This impact of nisin, however, faded during the later weeks of the trial, highlighting the importance of preventive measures rather than relying solely on the application of one antimicrobial. Nisin, similarly, was able to inhibit the multiplication of mesophilic background microbiota of the product earlier in the shelf-life study. We additionally observed that pressure-stressed phenotypes of *L. monocytogenes* and *L. innocua* have similar fate, proliferation, and sensitivity to nisin relative to wild-type *L. monocytogenes* and *L. innocua*. These results, thus, indicate that the survivors of high-pressure processing should be carefully considered in hurdle validation studies and in the development of food safety and/or Hazard Analysis and Critical Control Points (HACCP) plans. Our study investigated *L. innocua* as a surrogate for *L. monocytogenes* since the former is typically considered an avirulent microorganism for humans. Under the conditions of our experiment, we observed that *L. innocua* has comparable fate and proliferation trends during shelf-life and sensitivity to nisin and thus could be used interchangeably with *L. monocytogenes* in future validation studies with similar scope.

## Data Availability

The datasets of the current study can be obtained by contacting this study’s corresponding author with reasonable requests. A request could be submitted by obtaining the contact information from the Public Health Microbiology Foundation^SM^ at https://publichealthmicrobiology.education/ (accessed on 11 February 2025). The SAS codes used for statistical analyses in the current study were derived from no-cost and publicly available sources with needed modifications and can be obtained by contacting the study’s corresponding author with reasonable requests.
